# Supplementary material for: Exploring Large MAF Transcription Factors: Functions, Pathology, and Mouse Models with Point Mutations
Source: Genes (Basel). 2023 Sep 27;14(10):1883. doi: 10.3390/genes14101883 (PMC10606904; doi:10.3390/genes14101883)
Supplement: Supplementary file 1 [file genes-14-01883-s001.zip › genes-2601480-supplementary.pdf]

Table S1. Phenotypes of patients carrying the c-MAF mutation

Domain	Mutation	Phenotype	DOI
DNA-binding domain	R288P	・congenital cataracts<br>・iris hypoplasia	10.1093/hmg/11.1.33. [23]
	R288P<br>K297R	・pulverulent cataract<br>・cataracts with the<br>・microcornea<br>・iris coloboma<br>・congenital cataracts	10.1136/bjo.87.4.411. [24]<br>10.1002/ajmg.a.31126. [25]
	R288P	・reduction of vibratory sensation	10.1126/science.1214314. [26]
Transcriptional activation domain	S54L<br>T58A/I<br>P59L/H<br>T62R<br>P69R	・Aymé–Gripp syndrome	10.1016/j.ajhg.2015.03.001. [27]<br>10.1186/s12881-017-0414-7. [28]<br>10.1111/cge.13651. [29]<br>10.1002/ajmg.a.61299. [30]<br>10.1002/ajmg.a.62540. [31]

Table S2. Phenotypes of patients carrying MAFA mutations

Domain	Mutation	Phenotype	DOI
Transcriptional activation domain	S64F	・diabetes<br>・insulinomatosis	10.1073/pnas.1712262115. [43]

Table S3. Phenotypes of patients carrying MAFB mutations

Domain	Mutation	Phenotype	DOI
Transcriptional activation domain	S54W<br>P59L<br>T62I/P<br>T63L/R<br>S65I<br>S66C<br>S69L<br>S70L/A<br>P71S	・MCTO (multicentric carpotarsal osteolysis)<br>・FSGS (focal segmental glomerulosclerosis)	10.1016/j.ajhg.2012.01.003. [67]
DNA-binding domain	L239P	・Duane syndrome<br>・FSGS (focal segmental glomerulosclerosis)	10.1016/j.ajhg.2016.03.023. [68]
	R288P	impair the generation and/or inhibit to sustain<br>・abducens neurons<br>・inner ear<br>・podocytes in kidneys	10.1016/j.kint.2018.02.025. [69]
